# Evaluation of Developmental Toxicants and Signaling Pathways in a Functional Test Based on the Migration of Human Neural Crest Cells

**DOI:** 10.1289/ehp.1104489

**Published:** 2012-05-09

**Authors:** Bastian Zimmer, Gabsang Lee, Nina V Balmer, Kesavan Meganathan, Agapios Sachinidis, Lorenz Studer, Marcel Leist

**Affiliations:** 1 Doerenkamp–Zbinden Chair of *In Vitro* Toxicology and Biomedicine, University of Konstanz, Konstanz, Germany; 2 Institute for Cell Engineering, Department of Neurology and Neuroscience, Johns Hopkins University School of Medicine, Baltimore, Maryland, USA; 3 Center of Physiology, Institute of Neurophysiology, University of Cologne, Cologne, Germany; 4 Developmental Biology Program, Sloan–Kettering Institute, New York, New York, USA

**Keywords:** cell migration, developmental toxicity, mercury, neural crest, triazoles, valproic acid

## Abstract

Background: Information on the potential developmental toxicity (DT) of the majority of chemicals is scarce, and test capacities for further animal-based testing are limited. Therefore, new approaches with higher throughput are required. A screening strategy based on the use of relevant human cell types has been proposed by the U.S. Environmental Protection Agency and others. Because impaired neural crest (NC) function is one of the known causes for teratologic effects, testing of toxicant effects on NC cells is desirable for a DT test battery.

Objective: We developed a robust and widely applicable human-relevant NC function assay that would allow for sensitive screening of environmental toxicants and defining toxicity pathways.

Methods: We generated NC cells from human embryonic stem cells, and after establishing a migration assay of NC cells (MINC assay), we tested environmental toxicants as well as inhibitors of physiological signal transduction pathways.

Results: Methylmercury (50 nM), valproic acid (> 10 µM), and lead-acetate [Pb(CH_3_CO_2_)_4_] (1 µM) affected the migration of NC cells more potently than migration of other cell types. The MINC assay correctly identified the NC toxicants triadimefon and triadimenol. Additionally, it showed different sensitivities to various organic and inorganic mercury compounds. Using the MINC assay and applying classic pharmacologic inhibitors and large-scale microarray gene expression profiling, we found several signaling pathways that are relevant for the migration of NC cells.

Conclusions: The MINC assay faithfully models human NC cell migration, and it reveals impairment of this function by developmental toxicants with good sensitivity and specificity.

Gestational and early-life exposure to chemicals can result in developmental toxicity (DT). Experimental and epidemiological studies have shown that environmental agents may affect the developing peripheral and central nervous system (CNS) in animals and humans (Crofton et al. 2011; Grandjean and Landrigan 2006; Makris et al. 2009).

At present, neurodevelopmental disorders affect 3–8% of the children born in Western countries, and the National Academy of Sciences has estimated that 12% of children in the United States suffer from at least one mental disorder. Exposure to environmental chemicals has been identified as one of several risk factors facilitating or triggering such disorders (Hass 2006; van den Hazel et al. 2006). However, compelling epidemiological evidence is available for only a small number of chemicals and compounds such as lead, methylmercury (MeHg), arsenic, polychlorinated biphenyls (PCBs), valproic acid (VPA), and polybrominated diphenyl ethers (PBDEs). Furthermore, the number of different chemicals that have been tested in animals is rather limited. A list of about 100 chemicals with a likelihood of developmental neurotoxicity in animals has been compiled on the basis of available literature (Crofton et al. 2011). Only about 200 chemicals and pesticides have undergone testing according to the Organisation for Economic Co-operation and Development (OECD) Test Guideline 426 for animal-based developmental neurotoxicity testing (OECD 2007) or its precursor documents (Makris et al. 2009). The available comparative toxicity data for these compounds indicate that mammals are often particularly sensitive to this form of hazard compared to other forms of toxicity (Raffaele et al. 2010).

One-third of all human congenital birth defects are associated with neural crest (NC) cells and their derivatives (Trainor 2010). NC cells develop initially in parallel with CNS precursors and are found on top and on both sides of (dorso-lateral to) the neural tube. A key event in vertebrate development is the delamination of NC cells from the neural tube, the epithelial-to-mesenchymal transition of these cells, and their migration to target sites in the periphery, where they give rise to neurons and glial cells of the peripheral nervous system in addition to cranial bone and cartilage (Le Douarin et al. 2008). Genetic factors (Lee et al. 2009) and environmental chemicals or drugs, such as pesticides and anticonvulsants, have both been identified as causes for NC-related developmental defects (Di Renzo et al. 2007; Fuller et al. 2002; Menegola et al. 2000).

Animal-based testing of developmental neurotoxicity as specified by OECD Test Guideline 426 (OECD 2007) for example, is expensive and requires highly qualified personnel. The enormous resource requirements preclude testing even the most abundant industrial chemicals already marketed (Hartung and Rovida 2009). Moreover, the field of developmental toxicology has experienced examples of strong species differences in the past (Hawkins 1983; Nau 1986). Therefore, the U.S. Environmental Protection Agency (EPA) and the National Research Council (NRC) have recommended a new strategy for toxicity testing in the 21st century (NRC 2007; U.S. EPA 2009) based on a shift towards the use of human cell–based systems and other assays allowing a high throughput of chemicals and testing over large concentration ranges. A further element of the vision is the identification of pathways of toxicity (i.e., the accessibility of the chosen models to mechanistic studies) (Andersen et al. 2011).

Because human pluripotent stem cells can give rise to any differentiated cell type, they are a powerful tool that can be used to mimic human development *in vitro*. Both embryonic stem cell (ESC) lines and induced pluripotent cells have recently been used to generate NC cells (Lee et al. 2010). If such cells could be used for toxicological testing, new improved assays for DT would become feasible. This would complement previous successful efforts using neuronal cells derived from different types of stem cells to model DT in the CNS (Moors et al. 2009; Zimmer et al. 2011a, 2011b).

We therefore carried out this study to develop a test system for NC cell migration, based on human NC cells. The cells were generated from human ESC (hESC), and they were characterized in depth as to their genuine properties compared to other neural precursors. We were interested in identifying a functional end point that is relevant to the *in vivo* situation and susceptible to disturbance by chemicals. To evaluate the robustness of the test system and the feasibility of studies with reasonable throughput and precision, we tested several known toxicants and pathway-specific control substances. Our evaluation of NC cell migration yielded useful toxicological information in an area of DT that has received only limited attention until now.

## Materials and Methods

**Figure 1:**
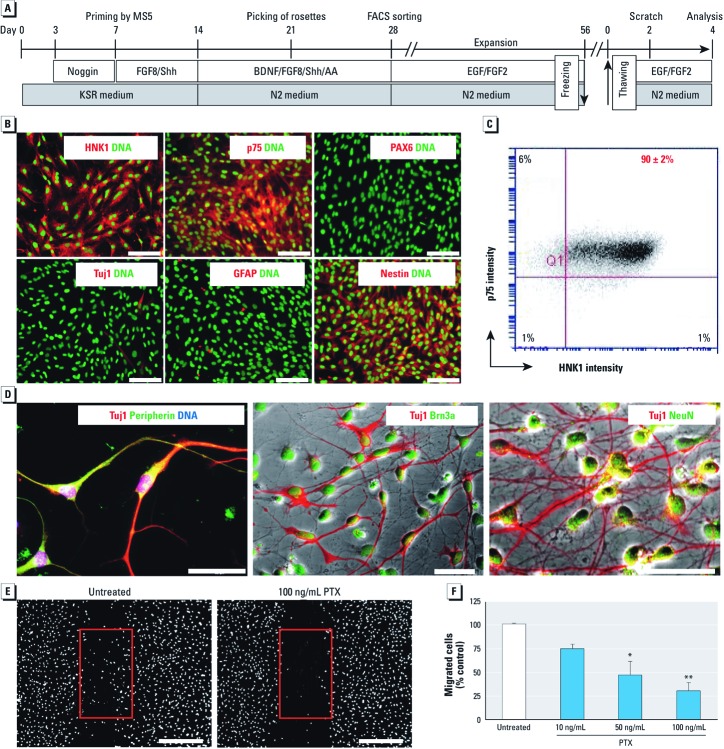
Characterization of hESC-derived NC cells. The schematic representation (*A*) illustrates the differentiation protocol and experimental procedures; AA, ascorbic acid; BDNF, brain-derived neurotrophic factor; FACS, fluorescence-activated cell sorting; FGF8, fibroblast growth factor-2; MS5, type of stromal cells; noggin, bone morphogenic protein antagonist; Shh, sonic hedgehog. (*B*) Immunocytochemical characterization of hESC-derived NC cells after thawing; bars = 200 µm; labels are color-keyed to images. (*C*) Flow cytometry analysis of NC cells for HNK1 and p75 expression. (*D*) Immunofluorescence analysis of peripheral neurons differentiated from NC cells; bars = 50 µm ; labels are color-keyed to images. (*E*) Representative images of cell migration in the absence or presence of pertussis toxin (PTX); bars = 500 µm. (*F*) Quantification of NC cell migration in the presence of PTX. The viability of the cells, as tested by resazurin reduction was 100 ± 5% under all conditions. *p < 0.05, and ** *p* < 0.01 compared with untreated controls.

*Cell culture and neural differentiation protocols.* The H9 hESC line was obtained from the Wisconsin International Stem Cell Bank (WISC Bank, Madison, WI, USA) and the isogenic reporter (GFP under the endogenous Dll1 promoter) cell line H9-Dll1 was provided by Mark Tomishima (Memorial Sloan–Kettering Cancer Center, New York, NY, USA). We carried out the importation of the cells and all experiments according to German legislation under license 1710-79-1-4-27 of the Robert Koch Institute (Berlin, Germany). Both cell lines were maintained on inactivated murine embryonic fibroblasts in medium supplemented with fibroblast growth factor-2 (FGF2). Differentiation into NC cells was initiated on MS5 stromal cells and continued as shown in Figure 1 and as described in Supplemental Material, p. 3 (http://dx.doi.org/10.1289/ehp.1104489). Differentiation towards CNS neuroepithelial precursor (NEP) cells was performed as described earlier (Chambers et al. 2009) and in more detail in Supplemental Material, p. 3. The HeLa 229, MCF-7, HEK 293, and 3T3 cell lines were cultured in Dulbecco’s modified Eagle medium (DMEM; Life Technologies, Carlsbad, CA, USA) supplemented with 10% fetal calf serum.

*Immunocytochemistry.* Cells were fixed directly on the cell culture plate. After incubation with the primary antibody overnight and with the appropriate secondary antibody, cells were stained with the DNA stain H-33342 and digitally imaged. For a detailed list of antibodies, see Supplemental Material, Table S1 (http://dx.doi.org/10.1289/ehp.1104489). We assessed cell proliferation using the Invitrogen Click-iT® EdU cell proliferation assay (Life Technologies) as described by the manufacturer.

*Flow cytometry analysis.* For flow cytometry analysis, cells were detached using accutase (PAA Laboratories GmbH, Pasching, Austria) and stained for 30 min on ice with antibodies specific for HNK1 (cell-surface glycoprotein) and p75 (low-affinity nerve growth factor receptor; LNGFR). After incubation with the appropriate secondary antibodies for 30 min on ice, cells were analyzed using a C6 flow cytometer (Accuri Cytometers, Inc., Ann Arbor, MI, USA). We processed and analyzed data using the Accuri CFlow Plus software, version 1.0.1727.

*Whole genome transcriptome analysis.* We isolated RNA from the cell cultures and prepared it for microarray hybridizations as described earlier (Wagh et al. 2011). We performed gene expression analysis as described in Supplemental Material, p. 4 (http://dx.doi.org/10.1289/ehp.1104489).

*Cell migration analysis.* Cell migration analysis was carried out using a scratch assay design as described by Lee et al. (2009) with minor changes. Briefly, a confluent layer of cells was scratched using a 20-µL pipette tip to create a cell-free gap. For some control experiments, culture inserts (Ibidi, Munich, Germany) were used to create a cell-free gap. The width of the cell-free gap was determined right after scratching the monolayer or removing the culture insert and used to define the region of interest. Then, the medium was removed and fresh medium containing the test chemicals was added. After 48 hr, a resazurin reduction assay was performed, and then fresh medium containing H-33342 (1 µg/mL) was added. After 30 min, random images along the scratch were taken at 4× magnification. We assessed the number of cells with H-33342-positive nuclei within the region of interest by manual counting.

*Chemical exposure during migration.* Cells were exposed to chemicals for 48 hr in N2 medium containing epidermal growth factor (EGF) (20 ng/mL) and FGF2 (20 ng/mL). After 48 hr exposure to chemicals, the cells were incubated with resazurin (10 µg/mL) in their cell culture medium for 60 min in order to determine viability. We analyzed resazurin reduction in cell culture medium fluorimetrically (λ_ex_ = 530 nm, λ_em_ = 590 nm), and values were normalized to untreated controls. [For a detailed list of chemicals and their tested concentration range see Supplemental Material, Table S2 (http://dx.doi.org/10.1289/ehp.1104489).]

## Results

*Characterization of hESC-derived NC cells.* A prerequisite for establishing a robust toxicological *in vitro* test system that can also be used in different laboratories is a protocol that allows production of large lots of identical cells. Moreover, only the ability to cyropreserve and to transport such cells allows for their broad applicability by laboratories not accustomed to the culture of hESC. To generate such a population of NC cells, we differentiated hESC as described earlier (Lee et al. 2010), and cryopreserved large batches after an additional phase of NC amplification in medium containing EGF and FGF2 (Figure 1A). We extensively phenotyped the thawed cells by immunostaining and expression analysis, and thawed cells were used for all further tests.

Immunofluorescence analysis showed a homogeneous expression of the specific NC markers AP2 [transcription factor AP-2 alpha (TFAP2A)] and SOX9 [SRY (sex determining region Y)-box 9] (data not shown) and HNK1 and p75, as well as the absence of the neuroepithelial marker PAX6 (paired box 6) (Figure 1B). The cells were positive for the general neuroectodermal marker nestin but not the Schwann cell marker glial fibrillary acidic protein (GFAP) or the neuronal marker (neuron-specific beta-III tubulin) Tuj1 (Figure 1B). Flow cytometry analysis confirmed the high purity of the expanded and cryopreserved NC culture, with > 97% of all cells expressing at least one of the surface markers HNK1 or p75 (Figure 1C). We also confirmed the NC properties, by differentiating the cells into peripheral neurons staining positive for beta-III tubulin (i.e., Tuj1) peripherin, Brn3a, and NeuN (Figure 1D).

*Characterization of the NC cell migration assay.* NC cells need to migrate to fulfill their biological function, and disturbance of this process by developmental toxicants leads to malformations. Therefore, we established an assay to test such interferences. Migration of NC (MINC) cells into a cell-free scratch area was followed for 48 hr with established methods (Lee et al. 2009). The variation of the scratch width was about 10% within and between experiments [see Supplemental Material, Figures S1A–C and S3A (http://dx.doi.org/10.1289/ehp.1104489)]. As an additional control for the potentially confounding effects of scratching, we used a system in which a cell-free gap was produced by a removable spacer without mechanical effects on cells or coating material. The gap width was 500 ± 50 µm, and cell migration was exactly the same as in the scratch assay (data not shown). To investigate the role of proliferation in our test system, we inhibited cell division by adding the cytostatic drug cytosine arabinoside (AraC; 10 µM). Under these conditions, DNA synthesis, as measured by incorporation of the thymidine analogue EdU, was completely inhibited, although the scratch was still repopulated to the same extent (see Supplemental Material, Figure S1D,E). Finally, we used pertussis toxin (PTX), which prevents receptor signaling via inhibitory G-proteins, as a pathway-specific positive control for MINC assay performance. NC cell migration was blocked concentration dependently by inhibition of this signal transduction mechanism, which is known to be important for cell migration (Figure 1E,F). For further exploration of signaling pathways relevant to the MINC assay, we tested eight different inhibitors that affect kinase signaling and actin polymerization. Six (positive control) compounds caused a significant inhibition, and two (negative controls) were inactive (see Supplemental Material, Figures S2 and S3). Thus, upon exposure to test chemicals, the MINC assay yielded quantitative data on the extent of disturbance of NC cell migration.

**Figure 2:**
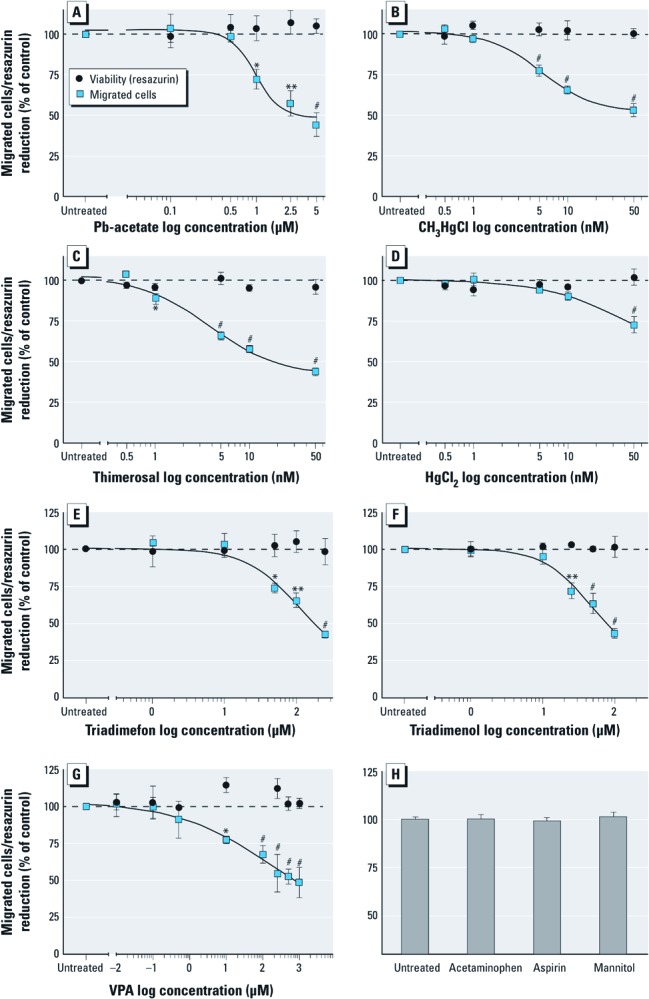
Inhibition of cell migration in NC cells by heavy metals and known NC toxicants shown by the effects of different compounds on NC cell migration and viability (resazurin assay). (*A*) Pb(CH_3_CO_2_)_4_. (*B*) CH_3_HgCl. (*C*) Thimerosal. (*D*) HgCl_2_. (*E*) Triadimefon. (*F*) Triadimenol. (*G*) VPA. (*H*) High concentrations (250 µM) of acetaminophen, aspirin, or mannitol did not alter NC cell migration. Data are presented as mean ± SD of at least three independent experiments normalized to untreated controls. * *p* < 0.05, ** *p* < 0.01, and ^#^ *p* < 0.001 compared with untreated controls.

*Inhibition of NC cell migration by toxicants.* After the initial evaluation of MINC assay performance, we examined the two general neurodevelopmental toxicants, lead and MeHg chloride (CH_3_HgCl). Pb-acetate [Pb(CH_3_CO_2_)_4_] reduced NC cell migration at concentrations ≥ 1 µM (72 ± 12%) (Figure 2A). CH_3_HgCl significantly inhibited NC cell migration at a concentration of 50 nM (53 ± 10%) in each of 13 independent assays (Figure 2B). These data indicate that the MINC assay has a high sensitivity for broad-acting developmental toxicants.

To further characterize the response dynamics of the MINC assay, we tested whether different mercurials may be ranked according to their potency. Thimerosal reduced NC cell migration at least as potently and effectively as CH_3_HgCl (lowest effective concentration in the range of 1–5 nM), whereas inorganic mercury (HgCl_2_) was about 10-fold less potent (Figure 2C,D). In all further tests of this study, CH_3_HgCl (50 nM) was measured in parallel with unknown compounds as a positive control. The acceptance criterion for the use of data from all experiments was a > 35% inhibition effect by MeHg. The average signal-to-noise ratio for this effect was 7.7, and the toxicity of MeHg was also observed under altered assay conditions, e.g., in the presence of the migration-enhancing medium supplement AlbuMax® [see Supplemental Material, Figure S3B (http://dx.doi.org/10.1289/ehp.1104489)].

In the next step, we investigated chemicals that are known to affect NC cell function during development. The triazole fungicides triadimefon and triadimenol are developmental toxicants that alter NC cell migration *in vivo* and *in vitro* (Di Renzo et al. 2007; Menegola et al. 2000; Papis et al. 2006). Both compounds triggered specific adverse effects in the MINC assay (in the absence of cytotoxicity) at the relatively low concentrations of > 50 µM (74 ± 6%, triadimefon) and > 25 µM (72 ± 9%, triadimenol (Figure 2E,F). The antiepileptic drug VPA is a human reproductive toxicant, and also has adverse effects on the NC of several species during early stages of development (Pennati et al. 2001). We found here that VPA inhibits NC cell migration at concentrations of ≥ 10 µM (77 ± 2%) (Figure 2G). When tested at concentrations of 250 µM, several other substances such as acetaminophen, aspirin, or mannitol did not show any effect at all (Figure 2H). Thus, the MINC assay detected known *in vivo* NC toxicants with high sensitivity, whereas supposedly innocuous chemicals gave no positive signal in the assay.

**Figure 3:**
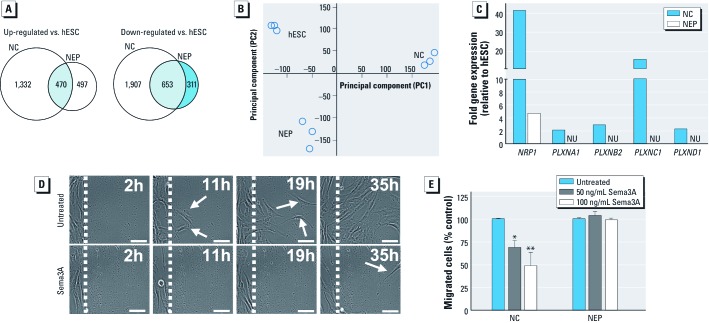
Transcriptome analysis of NC cells and specific migratory control. (*A*) Genome-wide transcription profiles were obtained for hESC, NC cells, and NEP cells; the number of significantly up-regulated or down-regulated genes is shown for NC cells and NEP cells relative to hESC. (*B*) Two-dimensional principal component analysis of the chip data; each circle indicates one experiment (*n* = 3 for each cell type). (*C*) Comparison of semaphorin receptor (NRP1, neuropilin 1; PLXN, plexin) expression in NC cells and NEP cells (NU, not up-regulated). (*D*) Migration of untreated or semaphorin 3A-exposed (Sema3A; 100 ng/mL) NC cells was recorded by video microscopy [see Supplemental Material, videos S1 and S2 (http://dx.doi.org/10.1289/ehp.1104489)]. Representative images are shown for four time points; white arrows indicate migrating cells; bars = 50 µm. (*E*) Quantification of NC cell and NEP cell migration in the presence of Sema3A.

*Distinction of hESC-derived NC cells from CNS neural precursors.* We used broad transcriptome profiling to further investigate the difference between NC cells and CNS NEP cells. We compared the mRNA expression profiles of NC cells, NEP cells, and the corresponding hESC. The 1,802 transcripts up-regulated in NC cells compared to hESC (1,332 transcripts up-regulated in NC cells only in addition to 470 transcripts that up-regulated in both NC cells and NEP cells) included classical NC markers such as snail homolog 2 (Drosophila) (*SNAI2*; 154-fold), *SOX9* (10-fold), and *AP2* (*TFAP2A*; 8-fold], whereas the transcripts up-regulated in NEP cells comprised expected genes such as *PAX6* (117-fold) and forkhead box G1 (*FOXG1*; 16-fold). Only 470 up-regulated transcripts were shared between NC cells and NEP cells. Among the 2,560 transcripts down-regulated in NC cells (1,907 transcripts down-regulated in NC cells only in addition to 653 transcripts that were down-regulated in both NC cells and NEP cells), characteristic pluripotency genes such as *SOX2* (165-fold), Nanog homeobox (*NANOG*; 110-fold), and Oct3/4 [POU class 5 homeobox 1 (*POU5F1*); 27-fold] were identified. These genes, except for *SOX2*, were also identified among the down-regulated genes of NEP cells (Figure 3A). Principal component analysis of the complete transcriptome sets revealed that NC cells and NEP cells differed drastically from one another, and also from hESC (Figure 3B). This confirmed that the cells used for the MINC assay represent a genuine cell population, clearly distinct from CNS neural stem cells.

Transcriptome analysis indicated that five semaphorin receptors (e.g., Nrp1: 22-fold; Plxnc1: 16-fold) were up-regulated (Figure 3C) in NC cells. Therefore, we tested whether the *in vivo* repellent ligand semaphorin 3A (Sema3A) inhibits NC cell migration. Time-lapse video microscopy showed the normal migration behavior of NC cells, and the strongly arresting effect of Sema3A on the cells (Figure 3D) [see also Supplemental Material, Videos S1 and S2 (http://dx.doi.org/10.1289/ehp.1104489)]. Quantification showed a concentration dependent inhibition for NC cells (Figure 3E).

**Figure 4:**
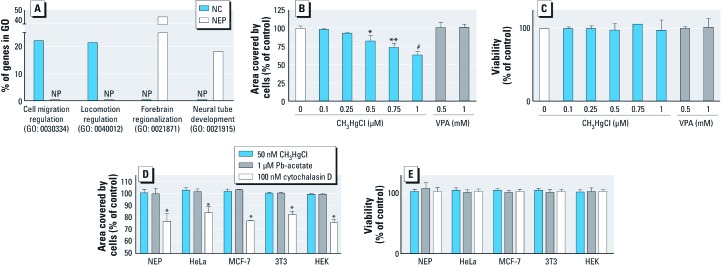
Migration capacity and its modulation in different cell types. (*A*) Examples for gene ontologies with strongly differential expression in NC cells and NEP cells. The four chosen GOs contained on average 170 genes. The fraction of genes identified to be up-regulated is indicated; NP, not present. (*B,C*) Effect of increasing concentrations of CH_3_HgCl and VPA on NEP cell migration and cell viability. (*D,E*) Quantification of cell migration and cell viability of four different cell lines and NEP cells in the presence of MeHg, Pb(CH_3_CO_2_)_4_, and cytochalasin D; data are normalized to respective untreated controls (set to 100%) and presented as mean ± SD of three independent experiments. * *p* < 0.05, ** *p* < 0.01, and ^#^ *p* < 0.001 compared with untreated controls.

*Cell type comparisons concerning compound effects on migration.* Many cell types are capable of migrating and this process also plays an important role in the CNS. In the present study, we compared the relative importance in the gene expression programs of NC cells and NEP cells. For this analysis, gene ontologies (GOs) that were statistically overrepresented among the genes up-regulated in the respective cell types were identified with bioinformatic tools. Strikingly, genes belonging to 18 GOs associated with migration [see Supplemental Material, Table S3 (http://dx.doi.org/10.1289/ehp.1104489)] and several hundred genes involved in cell motility were up-regulated in NC cells. The identification of 5 GOs associated with cell migration in NEP cells indicates the capability of this population for migration. However, other functions appeared to be more dominant. For instance, GOs selectively associated with NEP cells were “neural tube development” and “forebrain regionalization” (Figure 4A). Moreover, we directly identified genes that were differentially expressed in NC cells relative to NEP cells. Within the genes that were expressed more highly in NC cells, 30 GOs associated with migration were overrepresented (see Supplemental Material, Table S4). However, overrepresentation of the GO “locomotion” in genes more highly expressed in NEP cells again suggested that not only are these cells also migratory, but their movement behavior may be controlled by mechanisms that differ from those of NC cells. This hypothesis is supported by, for instance, largely different patterns of integrin expression. Whereas the genes integrin α8 (*ITGA8*), *ITGA4*, and *ITGA11* were up-regulated in NC cells only, *ITGB6* was up-regulated in NEP cells only (relative to hESC). Of all the regulated integrin genes identified, only integrin αV (*ITGAV*) and *ITGB8* were up-regulated in both cell populations (see Supplemental Material, Figure S4).

Because CH_3_HgCl is well known to inhibit cell migration in the CNS of rodents (Sass et al. 2001) and in human neural stem cells *in vitro* (Moors et al. 2009), we chose to quantify the effects of this compound on the migration of NEP cells. We confirmed the published findings that CH_3_HgCl inhibits CNS neural precursor migration at concentrations of ≥ 500 nM (82 ± 8%) (Figure 4B), that is, at an order of magnitude higher concentrations than in NC cells tested in the same setup (Figure 2B). We also examined the effects of VPA. This established human teratogen is known to affect neural tube closure, but data indicating its effects on migration of CNS neural precursors are scarce. Athough VPA inhibited NC cell migration in the 10–100 µM range (Figure 2G), it had no effect on NEP cell migration even at concentrations of 1 mM (Figure 4B). Corresponding cytotoxicity data are shown in Figure 4C. Thus, findings with these two developmental neurotoxicants indicated that NC cell migration is affected differently by chemicals than is NEP cell migration.

For a broader comparison of the effect of chemicals on the migration of NC cells versus other cell types, we chose the human cancer cell lines HeLa and MCF-7, the human nontransformed embryonic kidney cell line HEK293, and the mouse fibroblast cell line 3T3 in addition to NEP cells. All these cells migrated in the scratch assay [shown for HEK293 cells, see Supplemental Material, Video S3 (http://dx.doi.org/10.1289/ehp.1104489)], and their function was inhibited by cytochalasin D. However, none of these cells reacted to the low metal concentrations that inhibited the MINC assay (Figure 4D). Corresponding cytotoxicity data is shown in Figure 4E. These results suggest that the MINC assay specifically detects impairments of cell function that are not detectable by other assay systems. Moreover, NC cells appeared to be more susceptible to the inhibition of migration than various other cell types (Figures 2 and 4A,C). These results indicate that the MINC assay is highly sensitive to developmental toxicants and that it may yield information on their relative hazard.

## Discussion

NC cells generate a large number of different cell types all over the body. Therefore, impairment of their migration can cause multiple developmental defects (Ferretti 2006). In the present study, we describe a new approach to identify NC toxicants, based on human NC cells differentiated from hESC. Following the procedure suggested for developmental neurotoxicity assay development (Crofton et al. 2011), we used the MINC assay to test > 20 compounds, including negative controls, end point–specific controls, general developmental neurotoxicity compounds, and chemicals known to specifically impair NC cell migration *in vivo*. This yielded information on performance parameters of the assay as well as on underlying signaling pathways. Concerning the response dynamics of the MINC assay, we found at least one substance, the protein preparation AlbuMax® (Life Technologies), that increased migration. Thus, the MINC assay may detect compounds that inhibit and compounds that accelerate NC cell migration.

Impairment of NC development and function has been observed in different vertebrate test systems, using either *Xenopus*, zebrafish, chicken, or rodents and for a variety of chemicals ranging from fungicides and anticonvulsant drugs to PCBs (Di Renzo et al. 2007; Fuller et al. 2002; Grimes et al. 2008; Menegola et al. 2000; Papis et al. 2006). The triazole fungicides triadimefon and triadimenol have been shown in rats and *Xenopus* embryos to induce cranio-facial malformations, which were associated with abnormal NC cell migration. In cultured chicken embryo neural tube segments, treatment with the anticonvulsant VPA not only decreased the number of migrating NC cells, but the treatment also changed the type of NC cell migration from individually migrating cells to migration as an epithelial sheet. (Di Renzo et al. 2007; Fuller et al. 2002; Papis et al. 2006). Whether these changes in the type of migration can also be found in human NC cells needs to be verified in further studies. In addition to the above compounds known to affect NC cells *in vivo* and *in vitro*, we also tested the general developmental neurotoxicants CH_3_HgCl and Pb(CH_3_CO_2_)_4_, which have not been characterized for their effects on NC cells. The high potency of these chemicals in the MINC assay suggests that human NC cells may be a potential target of toxicity.

The concentration–response curves generated for all compounds allow estimates of their lowest observed effect levels (LOELs). Alternatively, a reference point for the classification of toxicity may be calculated by procedures similar to those used for the benchmark dose approach *in vivo*. The possibility to derive such LOELs has two interesting implications for the use of the MINC assay in hazard assessment. First, the MINC assay may be used for ranking of the relative hazard within a group of related compounds. Quantitative comparisons of chemicals in one assay system can already give valuable information when compounds are compared, for example, in read-across procedures as used in the European Union chemicals regulation law Registration, Evaluation, Authorization, and Restriction of Chemicals (REACH) or in the preselection of drug candidates for further development. For instance, inorganic and organic mercury compounds showed distinct toxicity thresholds in the MINC assay, similar to other model systems (Graeme and Pollack 1998; Moors et al. 2009). Thimerosal triggered toxicity at a higher, or at least a comparable, potency as MeHg. This indicates a very high hazard potential for this compound. However, more experiments and a careful comparison of the results with literature data will be required to evaluate the ability of the MINC assay to rank order compounds on their ability to predict *in vivo* potency with respect to developmental neurotoxicity.

Second, the LOELs may be used as point-of-departure for an *in vitro*–*in vivo* extrapolation of human adverse effects. In combination with exposure data, such information may contribute to a preliminary risk assessment of environmental chemicals to support prioritization of their further testing. Such reverse dosimetry, as also explored in the ToxCast program, may suggest different human equivalent doses (Wetmore et al. 2011), depending on the *in vitro* assay used. For instance, neuronal differentiation is affected at low nanomolar levels of CH_3_HgCl (Zimmer et al. 2011b), just as in the MINC assay, whereas attenuation of human neurite outgrowth (Stiegler et al. 2011), and inhibition of CNS neural stem cell migration (Moors et al. 2009) requires significantly higher concentrations.

Different types of migration may involve different toxicity pathways. Video microscopy indicated a mesenchymal type of cell migration for the NC cells. This type of migration was characterized by searching movements of growth-cone–like cellular processes of individual cells, and it differed strongly from the movement pattern of transformed cell lines, which migrated as an entire gliding front resembling more an epithelial-sheet amoeboid-like cell migration (Friedl and Wolf 2010; Rorth 2009). In CNS neural stem cell assays, migration is always associated with differentiation (Moors et al. 2009). In contrast to this, the MINC assay allowed the quantification of migration independent of differentiation, and also with negligible effects of proliferation on the readout. Such detailed biological information seems important for the interpretation of assay results. To test directly whether the type of neural cell used affected the test result, we differentiated hESC into CNS precursor cells using an established protocol (Chambers et al. 2009). The obtained NEP cells were initially compared with NC cells by genome-wide transcriptome analysis. This indeed provided genetic support for differential expression of migration-relevant genes. Subsequently, we addressed the functional differences. The MINC assay data corroborated the transcriptome results, as the NC cell repellant Sema3A inhibited NC cell migration, but did not affect NEP cell migration.

To obtain more data on toxicological differences between NC cells and NEP cells, we also compared the effects of chemicals on their migration. CH_3_HgCl was chosen because it is well established that it inhibits the migration of CNS progenitor cells *in vivo* and *in vitro*. We confirmed with NEP cells the published *in vitro* findings that such effects can be observed at concentrations of several hundred nanomolar (Moors et al. 2009). The MINC assay yielded qualitatively similar data, but sensitivity was 1–2 orders of magnitude higher. We selected VPA for comparison because this compound affects brain development by changes of neurogenesis and neuronal differentiation (Cowden et al. 2012; Hao et al. 2004) rather than by affecting migration. Accordingly, treatment of NEP cells with VPA did not alter the migratory potential of these CNS precursor cells, although the drug had potent effects in the MINC assay. These data demonstrate that different system and cell types are needed to create a full toxicological profile of a chemical. This also holds true when the systems measure apparently similar biological processes, such as migration. For instance, VPA enhances migration of mesenchymal stem cells and may show varying inhibiting or enhancing effects in different glioma cells (Knupfer et al. 2001; Tsai et al. 2010). Therefore, we believe that the use of genuine human NC cells for the MINC assay closes an important gap in test batteries assessing the many aspects of developmental toxicity.

A future perspective for the MINC assay may be the modeling of human genetic variability by using induced pluripotent stem cells as source material (Lee et al. 2010). Even higher throughput is allowed by specific migration plates developed by Platypus Technologies (Gough et al. 2011). A defined and reproducibly positioned gap in the cell layer is produced either by cell seeding stoppers (96-well plates) or by a biocompatible gel (384-well plates). Removal of the material starts the assay, and analysis can be performed either with high content imagers or with specialized fluorescence readers. Moreover, the 384-well format is fully compatible with automated liquid handling systems. A technical adaptation to such readouts, together with a closer characterization of the biological foundations of the assay, will assist the future mapping of toxicity pathways and the screening of larger panels of compounds. Together, this may contribute to an improved knowledge on the potential hazard of drugs and environmental toxicants to humans.

## Supplemental Material

(1.1 MB) PDF

Video S1 (MOV) **Transcript:** Migration analysis of untreated NC cells. Right after scratching, NC cells were imaged for 48 h as described in material and methods. The movie runtime of 1 min 22 sec corresponds to 48 h real time.

Video S2 (MOV) **Transcript:** Migration analysis of NC cells treated with 100 ng/ml semaphorin3A. Right after scratching, NC cells were imaged for 48 h as described in material and methods. The movie runtime of 2 min 45 sec corresponds to 48 h real time.

Video S3 (MOV) **Transcript:** Migration analysis of untreated HEK293 cells. Right after scratching HEK293 cells were imaged for 48 h as described in material and methods. The movie runtime of 1 min 16 sec corresponds to 48 h real time.
