# Supplementary material for: Evaluation of Developmental Toxicants and Signaling Pathways in a Functional Test Based on the Migration of Human Neural Crest Cells
Source: Environ Health Perspect. 2012 May 9;120(8):1116–22. doi: 10.1289/ehp.1104489 (PMC3440079; doi:10.1289/ehp.1104489)
Supplement: (1.1 MB) PDF [file ehp.1104489.s001.pdf]

Supplemental Material

Evaluation of Developmental Toxicants and Signaling Pathways in a Functional Test Based on the Migration of Human Neural Crest Cells

Bastian Zimmer[1], Gabsang Lee[2], Nina V. Stiegler[1], Kesavan Meganathan[3], Agapios Sachinidis [3], Lorenz Studer[4] and Marcel Leist[1]

[1]Doerenkamp-Zbinden Chair of *in vitro* Toxicology and Biomedicine, Department of Biology, University of Konstanz, D-78457 Konstanz, Germany

[2]Institute for Cell Engineering, Department of Neurology and Neuroscience, Johns Hopkins University School of Medicine, 733 N. Broadway, Baltimore, MD 21205, USA

[3]Center of Physiology, Institute of Neurophysiology, University of Cologne, D-50931 Cologne, Germany

[4]Developmental Biology Program, Sloan-Kettering Institute, 1275 York Ave, New York, New York 10065, USA.

Table of contents

- **Supplemental methods:** **page 3**
  1. Differentiation of human embryonic stem cells
  2. Cell culture of cell lines
  3. Flow cytometry analysis
  4. RNA isolation, Microarray labeling and hybridization
  5. Statistical filtration of significantly expressed genes
  6. Retrieving information on genes belonging to individual GOs
  7. Live cell video imaging of cell migration
  8. Statistics and data mining

- **Supplemental Material, Table S1** **page 6**
  Detailed list of antibodies used in this study

- **Supplemental Material, Table S2** **page 7**
  Detailed list of chemicals and growth factors used in this study

- **Supplemental Material, Table S3** **page 8**
  Significantly overrepresented GOs associated with migration identified by whole genome expression analysis of neural crest cells relative to hESC

- **Supplemental Material, Table S4** **page 9**
  Significantly overrepresented GOs associated with migration identified by whole genome expression analysis of neural crest relative to NEP

- **Supplemental Material, Figure S1** **page 10**
  Measurement of NC migration with a scratch repopulation assay

- **Supplemental Material, Figure S2** **page 11**
  Pharmacological modulation of NC migration

- **Supplemental Material, Figure S3** **page 12**
  Actin dynamics in migrating NC and response to a migration accelerating media supplement

- **Supplemental Material, Figure S4** **page 13**
  Integrin expression in NC and NEP

- **Supplemental Material, Videos S1, S2, S3** **page 13**

- **Supplemental References** **page 14**

Supplemental methods:

1. Differentiation of human embryonic stem cells

Differentiation of hESC to neural crest cells using the hESC line H9 or the isogenic reporter (GFP under the Dll1 promoter) cell line H9-Dll1 (Placantonakis et al. 2009) was performed exactly as described earlier in detail (Lee et al. 2010; Lee et al. 2007). Briefly, hESC were plated on a confluent layer of mitomycin C treated MS-5 stromal cells in KSR medium (DMEM supplemented with 15% serum replacement, 1x GlutaMax, non-essential amino acids (NEAA) and beta-mercaptoethanol, all ingredients from Invitrogen) (Lee et al. 2010). After 12 days of differentiation, medium was changed to DMEM/F12 supplemented with glucose, insulin, apo-transferrin, putrescine, selenite and progesterone as described (Lee et al. 2010) (from now on referred to as N2 medium), containing sonic hedgehog (SHH), fibroblast growth factor 8 (FGF8), brain-derived neurotrophic factor (BDNF) and ascorbic acid. Rosette structures were manually picked and harvested on day 21 of differentiation. The rosettes were then plated on previously poly-L-ornithine/laminin/fibronectin (PLO/L/FN) coated plates in N2 medium containing BDNF, SHH, FGF8 and ascorbic acid. After 7 days, cells were FACS sorted for positive expression of p75 (antibody obtained from Advanced targeting Systems) and HNK-1 (antibody obtained from Sigma). Appropriate secondary antibodies conjugated with PE and AlexaFluor647 were obtained from Invitrogen. Sorted cells were then expanded for 28 additional days in N2 medium supplemented with EGF (20 ng/ml) and FGF2 (20 ng/ml) (both R&D Systems Wiesbaden-Nordenstadt, Germany). Medium was changed every other day.

After 28 days of expansion, including 4-5 passaging steps, cells were detached from the plates using accutase (PAA, Pasching, Austria) and cryopreserved in 90% FCS 10 % DMSO. Cells were stored in liquid nitrogen. Expanded and cyropreserved cells were used for all further experiments.

Differentiation of NC cells into peripheral neurons was performed as described earlier (Lee et al. 2007). The cryopreserved cells were thawed and plated on PLO/L/FN coated plates at a density of 100 000 cells/cm^2 in N2 medium containing EGF and FGF2. After a 1 day attachment phase, cells were cultured in N2 medium containing different cytokines (Lee et al. 2010) for additional 3 weeks. Medium was changed every other day.

Differentiation of hESC to Pax6$^+$ neuroepithelial cells was performed as described earlier (Chambers et al. 2009) with minor changes. The initial noggin concentration was decreased to 35 ng/ml. Instead 600 nM dorsomorphin was added to complement for noggin.

2. Cell culture of cell lines

The HeLa229 (ATCC number: CCL-2.1), MCF-7 (ATCC number: HTB-22), HEK293 (ATCC number: CRL-1573) and 3T3 (ATCC number: CCL-92) cell lines were cultured in DMEM supplemented with 10% FCS and 2 mM GlutaMax at 37°C in a humidified atmosphere containing 5% CO_2. Cells were routinely passaged 3 times a week. The migration assay using these cell types was performed essentially as described for NC cells.

3. Flow cytometry analysis

For flow cytometry analysis, cells were detached using Accutase (PAA) and stained with HNK1 and p75 specific antibodies for 30 min on ice. After incubation with the appropriate secondary antibodies for 30 min on ice, cells were analyzed using an Accuri C6 flow cytometer (Accuri Cytometers, Inc. Ann Arbor, MI USA). Data were processed and analyzed using the Accuri CFlow Plus software.

4. Microarray labelling and hybridization

For global transcriptional profiling, the total RNA was isolated from neural progenitor cells using Trizol (Invitrogen, Damstadt, Germany), and purified with Qiagen RNeasy mini kits (Qiagen, Hilden, Germany). On column DNase digestion was performed as per the manufacturer's protocol. Before microarray analysis, the RNA was quantified with a NanoDrop N-1000 spectrophotometer (NanoDrop, Wilmington, DE, USA), and the integrity of RNA was confirmed with a standard sense automated gel electrophoresis system (Experion, Bio-Rad, Hercules, CA, USA). The samples were used for microarray analysis when the RNA quality indicator (RQI) number was higher than 8. For RNA amplification and biotin labelling, 100 ng total RNA were amplified for 16 h with Genechip 3' IVT Express Kit. After amplification, aRNA was purified with magnetic beads, and 15 µg of aRNA were fragmented with fragmentation buffer as per the manufacturer's instructions. 12.5 µg fragmented aRNA were hybridized with Affymetrix Human Genome U133 plus 2.0 arrays as per the manufacturer's instructions. The chips were placed in a GeneChip Hybridization Oven-645 for 16 h at 60 rpm and 45 ºC. For staining and washing, Affymetrix HWS kits were used on a Genechip Fluidics Station-450. For scanning, the Affymetrix Gene-Chip Scanner-3000-7G was used, and the image and quality control assessments were performed with Affymetrix GCOS software. All reagents and instruments were acquired from Affymetrix (Affymetrix, Santa Clara, CA, USA).

5. Statistical filtration of significantly expressed genes

Robust Multi-array Analysis was used for background correction and normalization. The raw dataset was transformed by Quantile normalization (Bolstad et al. 2003) with the R (Affy)-package (Gautier et al. 2004). MAS5 Expression Summary (Pepper et al. 2007) was used to detect present calls. Only 31567 probe sets out of 54613 received present calls as defined by the detection p-value of ≤ 0.05. Probe sets with "present" calls were selected and those with "absent" calls were eliminated.

One way Anova calculation was performed considering 'differentiation' as a factor with hESC as the defined control group. Moderated t-test calculation was applied for pairwise comparisons of NEP vs. hESC and NC vs. hESC. Differentially expressed transcripts were filtered with an FDR - controlled P value of ≤ 0.05 (95% confidence interval). A second filter selected for fold-change values. The Benjamini-Hochberg method was used to adjust the raw p-values to multiple testing and to reduce the false discovery rate.

Principal component (PC) analysis was performed using the Stats package in R. The first PC axis accounted for 37.4% of the variance in the data set of variable transcripts and the second accounted for 21.1%.

All microarray raw data and results have been deposited in a public database (GEO). [reference number to be added after manuscript acceptance]

6. Retrieving information on genes belonging to individual Gene Ontologies (GOs)

GOs often consist of 1000 genes or more. It is therefore difficult to display the genes of all Gene Ontologies analyzed in this study. We therefore, provide an easy web based approach to retrieve this information from an online database. The detailed procedure is described below.

Step 1: Open the webpage http://www.ensembl.org/index.html in your webbrowser
Step 2: Select BioMart in the top row of links
Step 3: Click on Dataset on the left side of the webpage
Step 4: Choose the "Ensembl genes 66" Database from the drop down menu

Step 5: Choose your Dataset of interest from the drop down menu. In this case “Homo sapiens genes”
Step 6: Now click on Filters (found on the left side of the webpage below “Dataset”)
Step 7: Now expand “Gene Ontology”
Step 8: Paste your GO term number of interest to the box “GO Term Accession". Alternatively you can use the respective GO term name and paste it into the box “GO Term Name”
Step 9: Now click on “Attributes” (found on the left side of the webpage below “Filters”)
Step 10: Expand “Gene” and choose which attributes you want to display. We recommend adding “Ensembl Gene ID”, “Description” and “Associated Gene Name”.
Step 11: Click on “Results” (located top left of the page)
Step 12: To remove potential duplicates within the list of genes, check the box “unique results only”
Step 13: Using the “View” dropdown menu, you can select the number of genes which are displayed. To see all the genes included in your GO of interest choose “All”.

7. Live cell video imaging of cell migration

Cells were seeded on 35 mm petri dishes (Ibidi GmbH, Munich, Germany) and treated as described above. Phase-contrast images from multiple predefined points (ROI) along the scratch were taken every 5 minutes for 48 h using a Nikon Biostation IM (Nikon GmbH, Duesseldorf, Germany) equipped with a 20x lens. Images were further processed and combined to video files using ImageJ. The width of an image frame is 240 µm.

8. Statistics and data mining

For the migration assay, the number of migrated cells was manually counted in ≥ 4 different fields per experiment. The untreated control fields contained 150 ± 44 (mean ± SD) migrated cells per field. In 13 independent experiments 672 ± 118 (means ± SEM) cells were counted for the untreated controls. All data displayed are means from independent biological experiments. Each biological experiment consisted of at least 3 technical replicates. Statistical differences were tested with GraphPad Prism 5.0 (Graphpad Software, La Jolla, USA) by applying ANOVA using Bonferroni's post-hoc test. Independent biological experiments (not technical replicates) were the basic unit used for statistical testing.

Supplemental Material, Table S1:
Detailed list of antibodies used in this study

**target protein/antibody name**	**dilution**	**catalogue number**	**provider**
Brn3a	1:500	AB5945	Millipore
GFAP	1:800	G3893	Sigma
HNK1	1:200	C6680	Sigma
Nestin	1:500	MAB1259	R&D
NeuN	1:200	MAB377	Millipore
P75	1:100	AB-N07	ATS*
Pax6	1:200	PRB-278P	Covance
Peripherin	1:200	SC-7604	Santa Cruz
Phalloidin-568 (Actin)	1:100	A12380	Invitrogen
Tubb3	1:1000	T2200	Sigma
Tuj1	1:1000	MMS-435P	Covance

* ATS: Advanced Targeting Systems

Supplemental Material, Table S2:
Detailed list of chemicals and growth factors used in this study

compound	concentration (range)	catalogue number	provider
Acetaminophen	250 µM	A7085	Sigma
AlbuMax® II	5%	11021	Invitrogen
Ara-C hydrochloride	10 µM	C6645	Sigma
Ascorbic acid	200 µM	A4034	Sigma
Ascorbic acid (migration assay)	250 µM	A4034	Sigma
BDNF	20 ng/ml	248-BD/CF	R&D
cAMP	1 mM	A9501	Sigma
CH_3HgCl	0.5 – 50 nM (5 nM)	442534	Sigma
CK-666	500 pM – 5 µM	182515	Calbiochem
CK-689	500 pM – 5 µM	182517	Calbiochem
cytochalasin D	1 – 100 nM	C8273	Sigma
D-Mannitol	1 µM – 1 mM	M1902	Sigma
Dorsomorphin	600 nM	3093	Tocris
EGF	20 ng/ml	236-EG	R&D
FGF2 (differentiation)	20 ng/ml	233-FB/CF	R&D
FGF2 (hESC culture)	10 ng/ml	13256-029	Invitrogen
FGF8	100 ng/ml	423-F8/CF	R&D
GDNF	20 ng/ml	212-GD/CF	R&D
$HgCl_2$	0.5 – 50 nM (50 nM)	203777	Sigma
Lead-acetate ($Pb(CH_3CO_2)_4$)	0.1 – 5 µM (1 µM)	398845	Sigma
Locostatin (UIC-1005)	500 pM – 5 µM	219469	Calbiochem
Locostatin neg. ctrl. (UIC-1017)	500 pM – 5 µM	219470	Calbiochem
NGF	10 ng/ml	256-GF/CF	R&D
Noggin	500 ng/ml	719-NG	R&D
NSC23766	10 nM – 5 µM	2161	Tocris
NT3	10 ng/ml	267-N3/CF	R&D
Pertussis toxin	50 -100 ng/ml	P2980	Sigma
PP2	0.5 – 1 µM (1µM)	P0042	Sigma
SB431542	10 µM	1614	Toocris
Semaphorin3A	50 – 100 ng/ml	1250-S3	R&D
Sonic Hedgehog (Shh)	20 ng/ml	1845-SH/CF	R&D
SP600125	0.5 – 10 µM	S5567	Sigma
Thimerosal	0.5 – 50 nM (1 nM)	T4687	Sigma
Triadimefon	1 – 250 µM (50 µM)	45693	Sigam
Triadimenol	1 – 100 µM (25 µM)	46138	Sigma
Valproic acid (VPA)	0.01 – 1000 µM	P4543	Sigma

The highest non-cytotoxic concentration, determined in a cell viability assay (resazurin reduction) after 48 h, was used as highest concentration for the NC cell migration assay. The high concentration indicated in the table corresponds to the highest non-cytotoxic concentration found in the pre-screening assay. Where concentration ranges are given for mercurial compounds or lead, the LOEL in the NC migration assay is indicated in brackets. Note the 10 - 50 fold differences between organic and inorganic mercury compounds.

Supplemental Material, Table S3:

Significantly overrepresented GOs associated with migration, identified by whole genome expression analysis relative to hESC

GO term	term domain and name	p-value
GO:0040011	locomotion	2.02e-14
GO:0040012	regulation of locomotion	2.87e-12
GO:0016477	cell migration	8.72e-12
GO:0042330	taxis	8.21e-11
GO:0006935	chemotaxis	8.21e-11
GO:0030334	regulation of cell migration	1.08e-11
GO:2000145	regulation of cell motility	1.77e-11
GO:0048870	cell motility	3.50e-10
GO:0040017	positive regulation of locomotion	6.62e-08
GO:0040013	negative regulation of locomotion	3.53e-07
GO:2000147	positive regulation of cell motility	5.06e-07
GO:0030335	positive regulation of cell migration	5.06e-07
GO:0043542	endothelial cell migration	9.30e-07
GO:0009611	response to wounding	1.22e-06
GO:0042060	wound healing	1.35e-06
GO:0030336	negative regulation of cell migration	3.65e-06
GO:2000146	negative regulation of cell motility	3.65e-06
GO:0010594	regulation of endothelial cell migration	1.25e-05

Whole genome mRNA expression in neural crest cells was analyzed using the affymetrix microarray platform. Gene expression was compared to gene expression in undifferentiated human embryonic stem cells. Significantly upregulated genes in NC cells were then further analyzed using the web-based gene onthologie (GO) analyzing tool g:Profiler (Reimand et al. 2007). Statistically overrepresented GOs dealing with cell migration are displayed. To display the genes belonging to each GO, the procedure described in the Supplemental method section allows easy access to this information.

Supplemental Material, Table S4:

Significantly overrepresented GOs associated with migration in NC vs. NEP, identified by whole genome expression analysis relative to NEP

GO term	term domain and name	p-value
GO:0040011	locomotion	1.99e-11
GO:0042330	taxis	2.90e-06
GO:0006935	chemotaxis	2.90e-06
GO:0051674	localization of cell	2.84e-10
GO:0030030	cell projection organization	1.34e-07
GO:0001837	epithelial to mesenchymal transition	5.30e-03
GO:0030029	actin filament-based process	2.83e-05
GO:0030036	actin cytoskeleton organization	2.28e-04
GO:0006928	cellular component movement	1.70e-08
GO:0048870	cell motility	2.84e-10
GO:0016477	cell migration	8.96e-11
GO:0043542	endothelial cell migration	5.53e-05
GO:0043534	blood vessel endothelial cell migration	1.62e-02
GO:0014812	muscle cell migration	2.54e-03
GO:0014909	smooth muscle cell migration	3.14e-03
GO:0060326	cell chemotaxis	1.03e-02
GO:0032879	regulation of localization	1.92e-04
GO:0051270	regulation of cellular component movement	9.65e-08
GO:0051272	positive regulation of cellular component movement	3.64e-05
GO:0040012	regulation of locomotion	1.69e-07
GO:0040017	positive regulation of locomotion	8.16e-06
GO:2000145	regulation of cell motility	1.74e-07
GO:0030334	regulation of cell migration	8.83e-07
GO:0010594	regulation of endothelial cell migration	4.97e-03
GO:0014910	regulation of smooth muscle cell migration	8.31e-03
GO:2000147	positive regulation of cell motility	3.27e-05
GO:0030335	positive regulation of cell migration	7.44e-05
GO:0010595	positive regulation of endothelial cell migration	3.53e-02
GO:0009611	response to wounding	3.04e-06
GO:0042060	wound healing	1.64e-06

Whole genome mRNA expression in neural crest cells was analyzed using the affymetrix microarray platform. Gene expression was compared to gene expression in NEP. Significantly upregulated genes in NC cells were then further analyzed using the web-based gene onthologie (GO) analyzing tool g:Profiler (Reimand et al. 2007). Statistically overrepresented GOs dealing with cell migration are displayed. To display the genes belonging to each GO, the procedure described in the Supplemental method section allows easy access to this information.

Supplemental Material, Figure S1:
Measurement of NC migration with a scratch repopulation assay

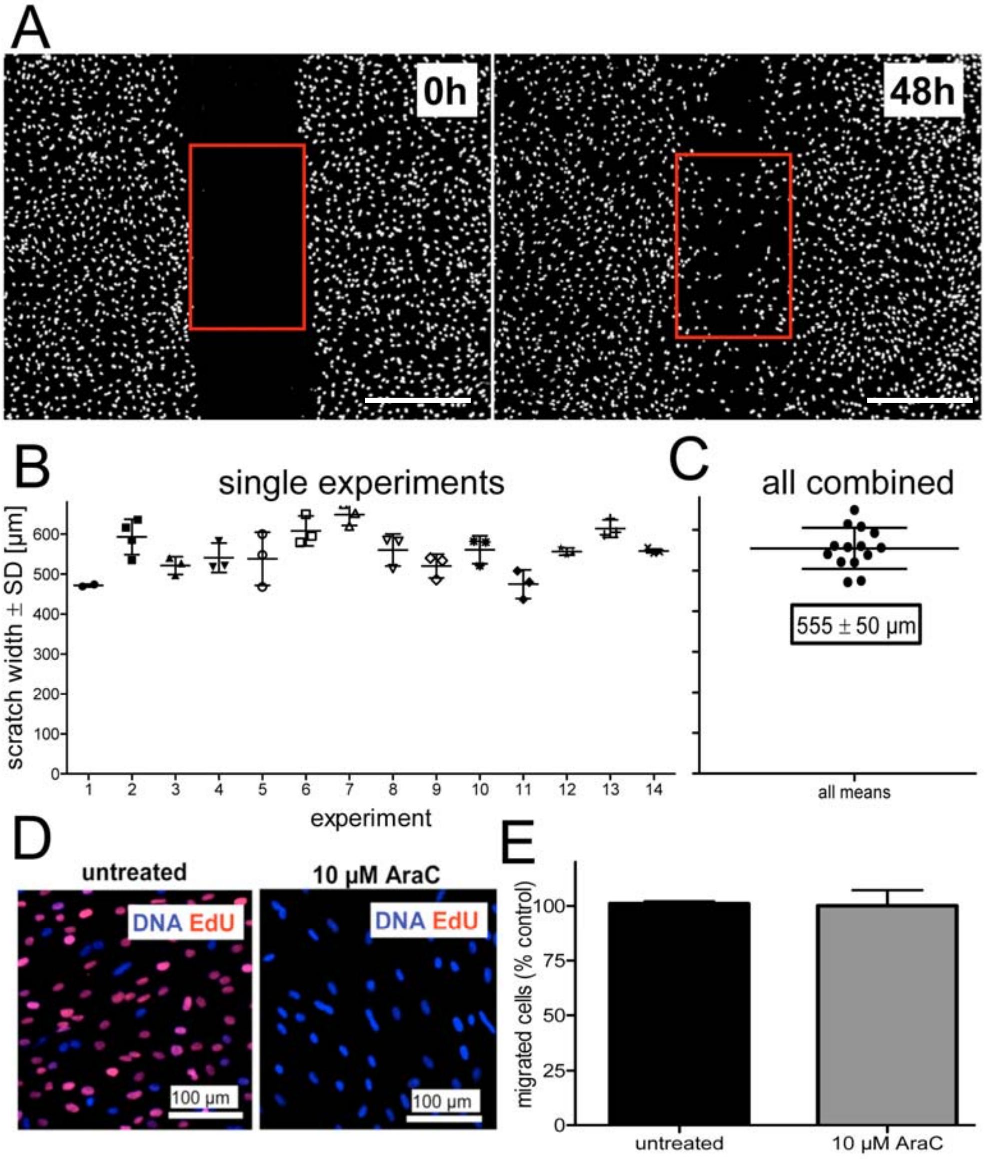

In a homogenous NC culture, cells were removed mechanically along an about 0.5 mm wide line using a pipette tip. Cells were visualized using the fluorescent nuclear stain H-33342. (A) Representative image of the scratch (0 h, left), used to define the region-of-interest (ROI) for quantification (red rectangle); a typical situation at the end of the assay is shown (48 h, right). Bars = 500 µm (B) Measurement of the scratch width in 14 independent experiments (means of 3 technical replicates ± SD). (C) Mean scratch width ± SD of the experiments shown in B. (D) Cell proliferation was measured in NC cells during the migration assay in the presence or absence of 10 µM cytosine arabinoside (AraC). Proliferating cells incorporated EdU, and are stained in red. EdU (10 µM) was present throughout the assay (48 h). Bars = 100 µm. (E) Cell migration assay in the presence of 10 µM AraC. Data are normalized to untreated controls and displayed as means ± SD of 3 independent experiments.

Supplemental Material, Figure S2:
Pharmacological modulation of NC migration

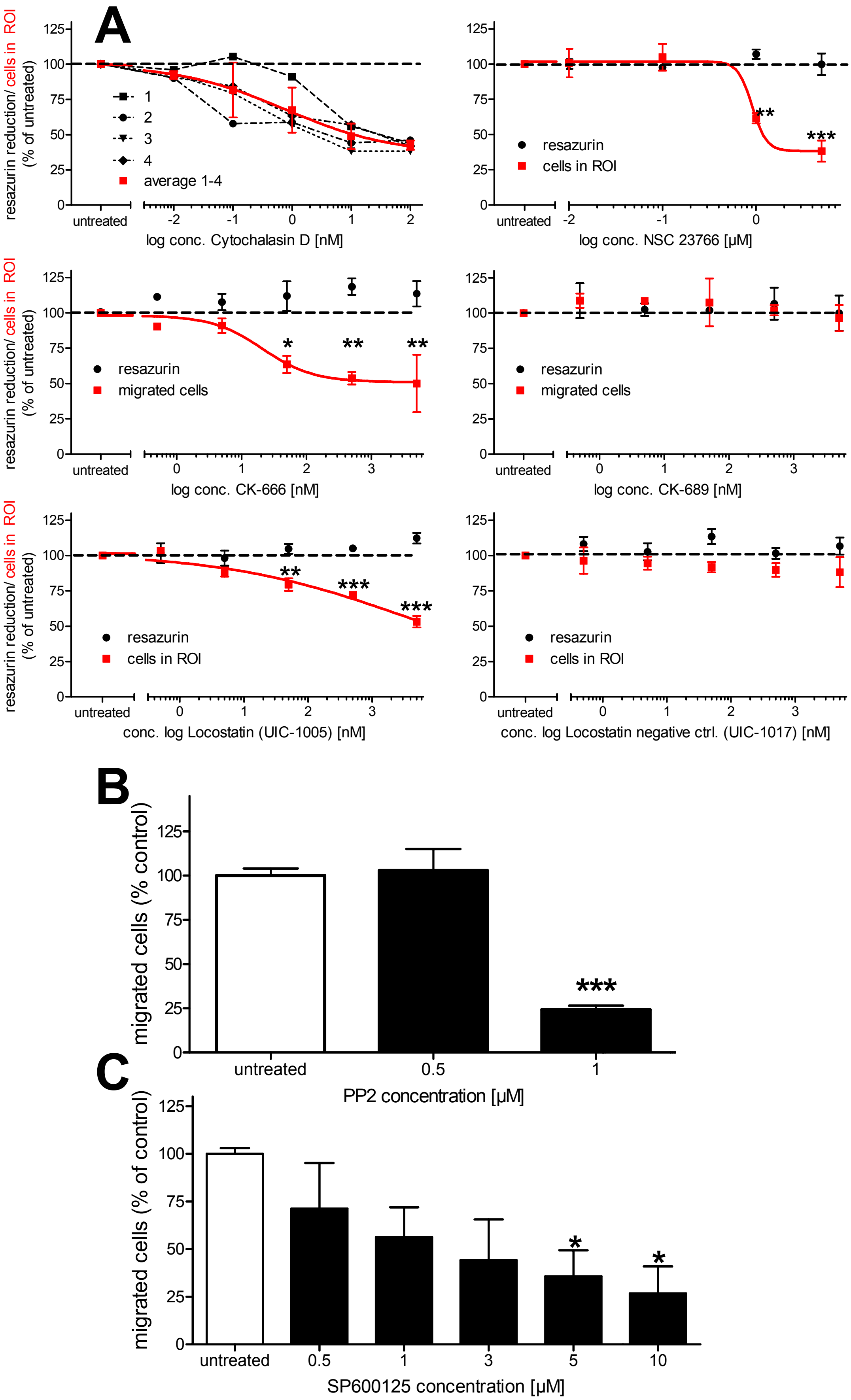

Supplemental Material, Figure S2: Positive and negative control compounds interfering with actin dynamics were tested in the MINC assay. (A) For cytochalasinD, 4 independent biological experiments are shown (dotted lines) to indicate variations of different experiments within the assay. (B) Neural crest cell migration was inhibited by 1 µM of the Src-family tyrosine kinase inhibitor PP2. (C) The selective inhibitor of c-Jun N-terminal kinase (JNK), SP600125 reduced cell migration in a concentration- dependent manner, with 5 and 10 µM of SP600125 reducing NC cell migration significantly. Note that no general cytotoxicity was observed in the resazurin assay (data not shown). Data are means ± SD of 3 independent experiments normalized to the untreated control. *: $p < 0.05$, **: $p < 0.01$, ***: $p < 0.001$.

Supplemental Material, Figure S3: Actin dynamics in migrating NC and response to a migration accelerating media supplement

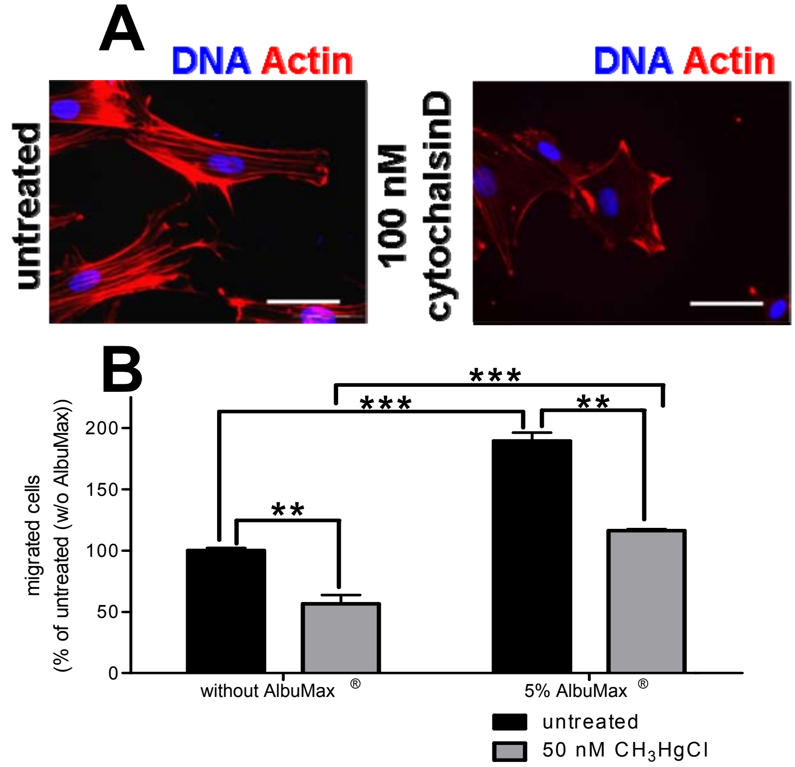

Supplemental Material, Figure S3: (A) Representative images of actin filaments in untreated migrating neural crest cells (left) and neural crest cells treated with 100 nM cytochalasin D (right). Actin filaments were visualized using phalloidin (red), H-33342 was used to counterstain nuclei (blue). Inhibition of migration with cytochalasinD correlated with a reorganization of F-actin. Stress fibres were lost, and only few cortical cytoskeletal structures remained. Bars = 50 µm. (B) The NC cell migration assay was performed in the presence of 5% AlbuMax® (the lipid rich version of bovine serum albumin). Incubation of migrating neural crest cells with AlbuMax® doubled cell migration (black bars). Methylmercury (grey bars) inhibited both the normal migration (without AlbuMax®) and the accelerated migration (with AlbuMax®) to the same extent (inhibition by 50 nM methylmercurychloride in the absence of AlbuMax® was about 57% compared to untreated cells. In the presence of AlbuMax®, NC cell migration was reduced by about 60%). Data are displayed as means ± SD of 2 independent biological experiments, each performed in triplicates. Data were normalized to untreated controls without AlbuMax®. **: $p < 0.01$, ***: $p < 0.001$.

Supplemental Material, Figure S4:
Integrin expression in NC and NEP

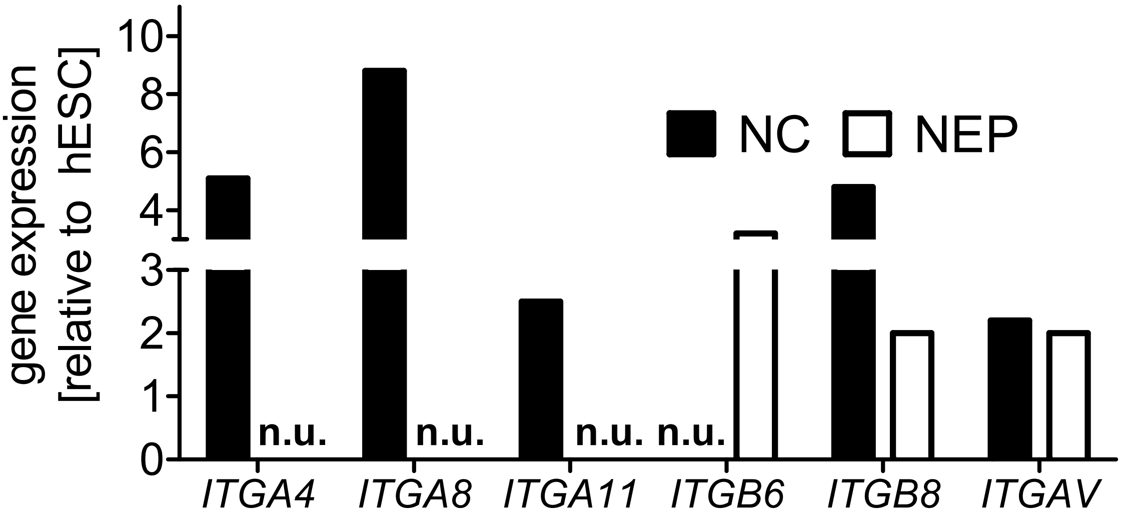

Comparison of integrin expression (*ITGA4*: integrin, alpha 4; *ITGA8*: integrin, alpha 8; *ITGA11*: integrin, alpha 11; *ITGB6*: integrin, beta 6; *ITGB8*: integrin, beta 8; *ITGAV*: integrin, alpha V) in NC and NEP relative to undifferentiated hESC. n.u.: not upregulated

Supplemental Material, Videos S1, S2, S3

The movie files have been uploaded to the EHP website. They may be considered as background information. Corresponding representative still images are found in figure 3. Supplemental Material Video S1, S2 and S3 are provided as separate .MP4 files.

Supplemental Material, Video S1
Migration analysis of untreated NC cells. Right after scratching, NC cells were imaged for 48 h as described in material and methods. The movie runtime of 1 min 22 sec corresponds to 48 h real time.

Supplemental Material, Video S2
Migration analysis of NC cells treated with 100 ng/ml semaphorin3A. Right after scratching, NC cells were imaged for 48 h as described in material and methods. The movie runtime of 2 min 45 sec corresponds to 48 h real time.

Supplemental Material, Video S3
Migration analysis of untreated HEK293 cells. Right after scratching HEK293 cells were imaged for 48 h as described in material and methods. The movie runtime of 1 min 16 sec corresponds to 48 h real time.

Supplemental References

Bolstad BM, Irizarry RA, Astrand M, Speed TP. 2003. A comparison of normalization methods for high density oligonucleotide array data based on variance and bias. Bioinformatics 19(2):185-193.

Chambers SM, Fasano CA, Papapetrou EP, Tomishima M, Sadelain M, Studer L. 2009. Highly efficient neural conversion of human ES and iPS cells by dual inhibition of SMAD signaling. Nat Biotechnol 27(3):275-280.

Gautier L, Cope L, Bolstad BM, Irizarry RA. 2004. affy - analysis of Affymetrix GeneChip data at the probe level. Bioinformatics 20(3):307-315.

Lee G, Chambers SM, Tomishima MJ, Studer L. 2010. Derivation of neural crest cells from human pluripotent stem cells. Nat Protoc 5(4):688-701.

Lee G, Kim H, Elkabetz Y, Al Shamy G, Panagiotakos G, Barberi T, et al. 2007. Isolation and directed differentiation of neural crest stem cells derived from human embryonic stem cells. Nat Biotechnol 25(12):1468-1475.

Pepper SD, Saunders EK, Edwards LE, Wilson CL, Miller CJ. 2007. The utility of MAS5 expression summary and detection call algorithms. BMC Bioinformatics 8:273.

Placantonakis DG, Tomishima MJ, Lafaille F, Desbordes SC, Jia F, Socci ND, et al. 2009. BAC Transgenesis in Human Embryonic Stem Cells as a Novel Tool to Define the Human Neural Lineage. Stem Cells 27(3):521-532.

Reimand J, Kull M, Peterson H, Hansen J, Vilo J. 2007. g:Profiler--a web-based toolset for functional profiling of gene lists from large-scale experiments. Nucleic Acids Res 35(Web Server issue):W193-200.
